# Upper extremity necrotizing fasciitis in a Covid-19 patient

**DOI:** 10.1080/23320885.2022.2028550

**Published:** 2022-01-21

**Authors:** Sriram Sankaranarayanan, Amanda F. Spielman, Anne-Sophie Lessard, Tarik Husain

**Affiliations:** a Department of Orthopaedics, NYU Langone Orthopedic Hospital/NYU School of Medicine, New York, NY, USA; b University of Miami, Miller School of Medicine, Miami, FL, USA; c Division of Plastic and Reconstructive Surgery, DeWitt Daughtry Department of Surgery, University of Miami, Miami, FL, USA; d Plastic, Orthopaedic and Hand Surgery at Mosa Surgery, Miami, FL, USA

**Keywords:** COVID-19, hypercoagulability, gangrene, necrotizing fasciitis, upper extremity

## Abstract

The novel COVID-19 virus has resulted in an immense burden in healthcare throughout the world. In addition to respiratory complications, COVID-19 has been associated with hypercoagulability and ischemic changes. We report a case of a patient with COVID-19 who presented with a rapidly progressing necrotizing fasciitis treated in our institution.

## Introduction

The novel Coronavirus disease 2019 (COVID-19) is caused by Severe Acute Respiratory Syndrome Coronavirus 2 (SARS-CoV-2). Although it is known for its respiratory complications, medical literature has shown that individuals with COVID-19 tend to have a spectrum of clinical manifestations, which includes coagulation abnormalities. Hyper-coagulability can worsen prognosis secondary to increased risk of pulmonary embolism, venous thrombo-embolism, limb ischemia, and digital ischemia. The underlying pathophysiology may be related to a severe inflammatory response leading to cytokine induced endothelial damage, micro-vascular thrombosis, and the development of pro-thrombotic anti-phospholipid antibodies [1–3]. Elevated D-dimer has been correlated with illness severity and increased mortality [4].

The COVID-19 pandemic has caused substantial disruptions to health care delivery. Initial reports from China have helped us understand the approaches to the management of the infectious complications of COVID-19. An early case series from intensive care units in Wuhan, China described seven patients with peripheral ischemia and elevated D-dimer levels suggesting hypercoagulability [2]A more recent study presented two cases of digital ischemia in ICU patients with COVID-19 [5]. There appears to be a relationship between hyper-coagulation and an overall poor clinical prognosis in COVID-19 patients [5].

Necrotizing fasciitis is a rare condition characterized by widespread necrosis of subcutaneous tissue and fascia. The mortality rate with necrotizing fasciitis has been reported to be as high as 32% [6]. There are several studies in literature that show that early identification and management of necrotizing fasciitis is critical in reducing over-all mortality rates [6].

We present a case of a patient who was seen in our hospital with finger ischaemia, gangrene and necrotizing fasciitis of his hand and forearm with confirmed COVID-19. To our knowledge, this is the first case of necrotizing fasciitis of the upper extremity in a patient with confirmed COVID-19 being reported in literature.

## Case report

A 72-year-old male with past medical history of uncontrolled insulin dependent diabetes mellitus (Admission HbA1C of 15.5%), chronic Stage 4 kidney disease, congestive heart failure, peripheral vascular disease and previous foot amputation presented to the emergency department (ED) with complaints of severe progressive pain, swelling and discoloration of the ring and small fingers that started ten days ago. The patient denied a history of trauma to his hand. Upon arrival to the ED, patient’s vital signs included a temperature of 98.7 F, pulse rate of 66/min, respiratory rate of 16/min, and blood pressure of 87/47.

**Figure 1.**
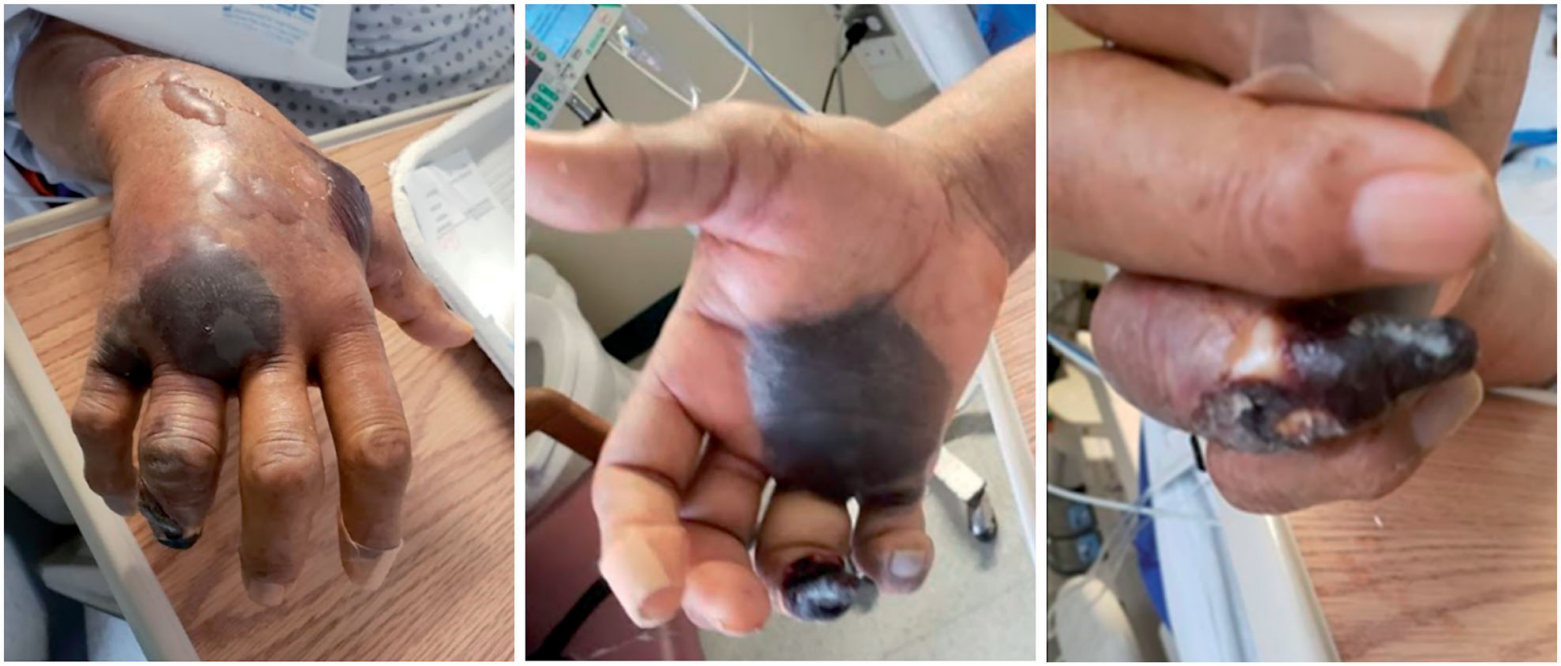
Presentation on admission.

While in the ED, physical examination of the patient’s hand revealed a wet gangrene of the right ring and small fingers. In addition, there were hemorrhagic blisters present over the hand as well as over the dorsal forearm (Figure 1). His labs were significant for WBC 28.93, ESR 65(reference normal of <20), CRP of 340 mg/L (reference normal of <10 mg/L), lactate 2.8 mmol/L (reference normal of < 2 mmol/L), Na+ 127 mEq/L, Hb 7.7 g/dL, creatinine of 2.65 mg/dL, glucose of 338 mg/dL, and a PT/INR of 13.5/1.2, D-Dimer of 1446 ng/mL (reference normal of <250 ng/mL). An arterial duplex revealed patent subclavian, axillary, radial and ulnar arteries. The clinical findings were suggestive of necrotizing fasciitis of the hand and forearm.

**Figure 2.**
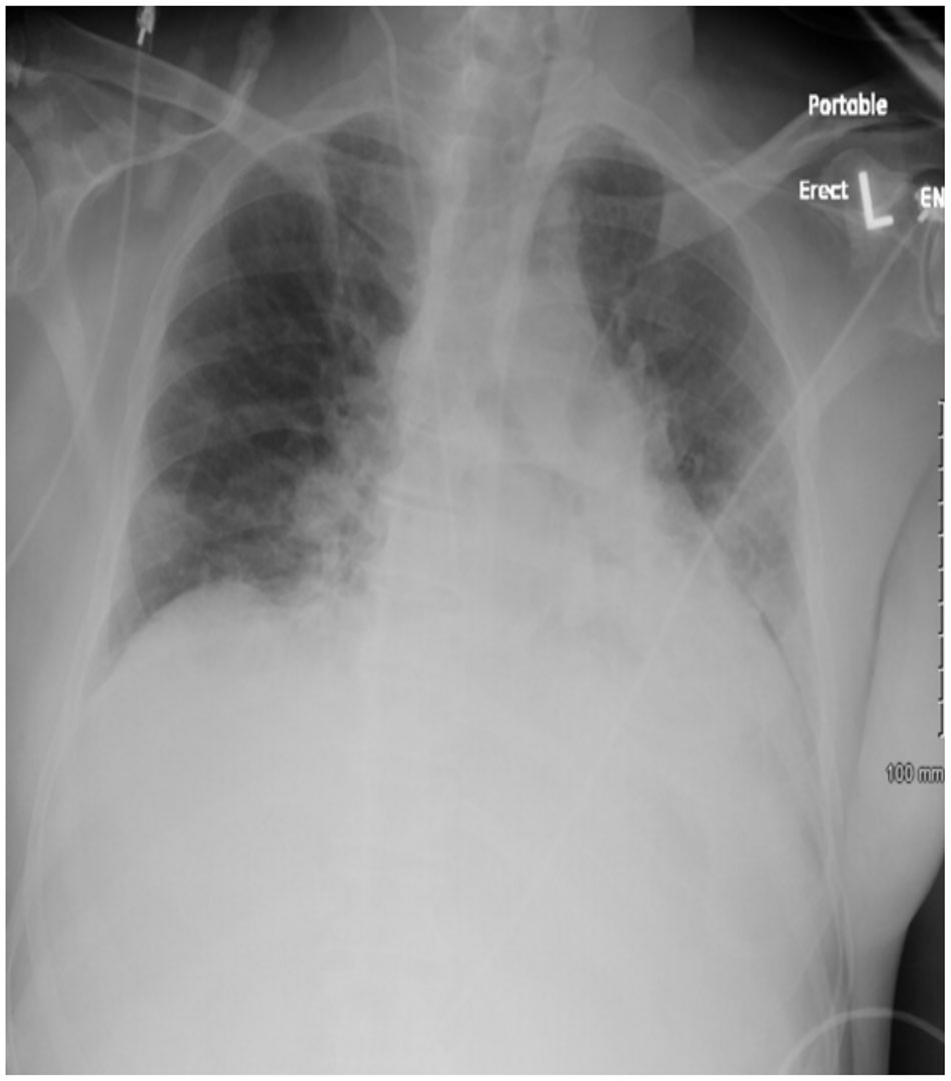
Chest Xray on admission.

A chest Xray obtained at the time of admission demonstrated patchy bilateral infiltrates as well as airspace decrease more on the left side than the right, a pattern that is seen with COVID-19 (Figure 2). At the time of admission, the patient did not require intubation. The Polymerase Chain Reaction testing for COVID-19 antigen was positive. He was able to maintain his oxygen saturation with 4 litres of nasal cannula 100 percent oxygen and supportive therapy.

**Figure 3.**
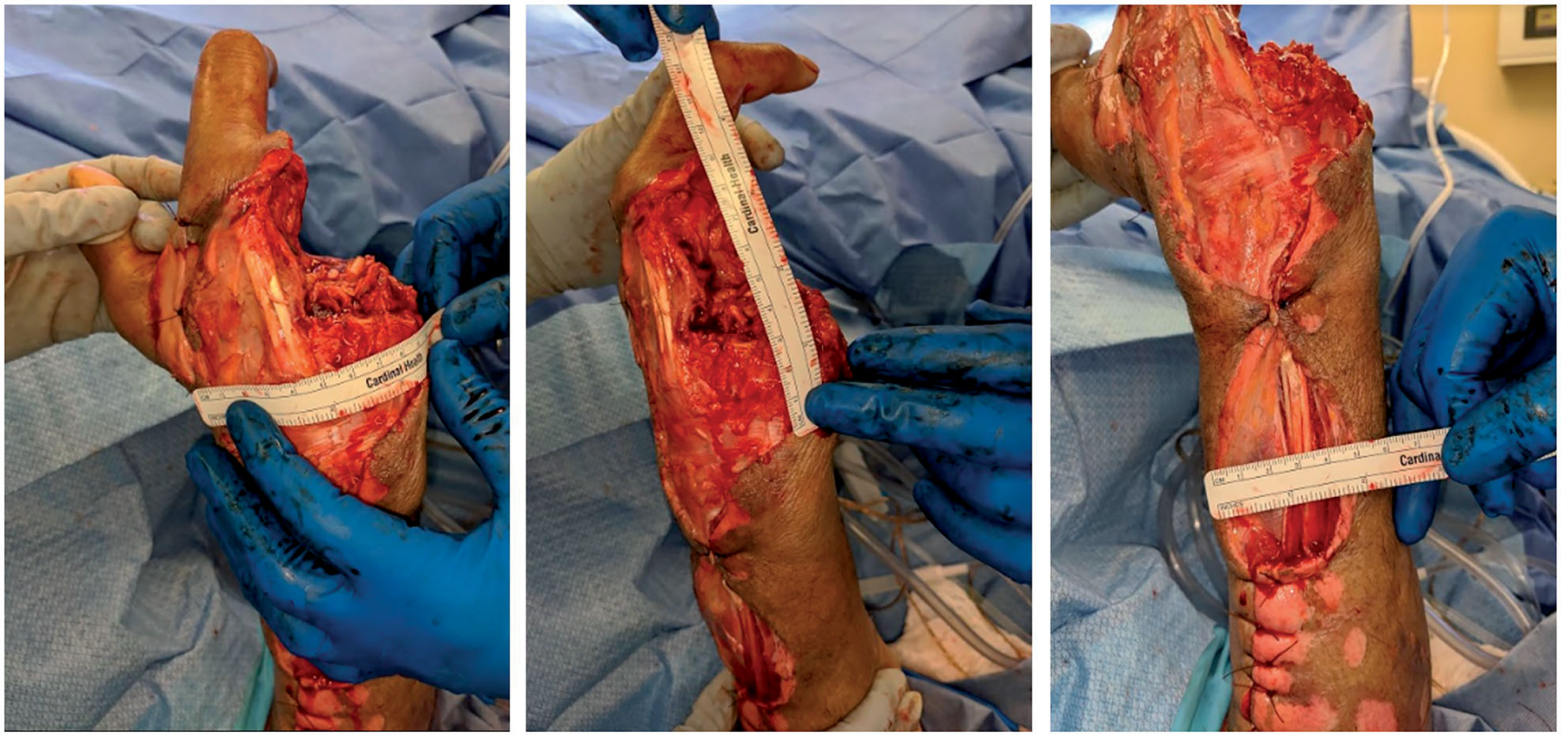
After serial debridements.

**Figure 4.**
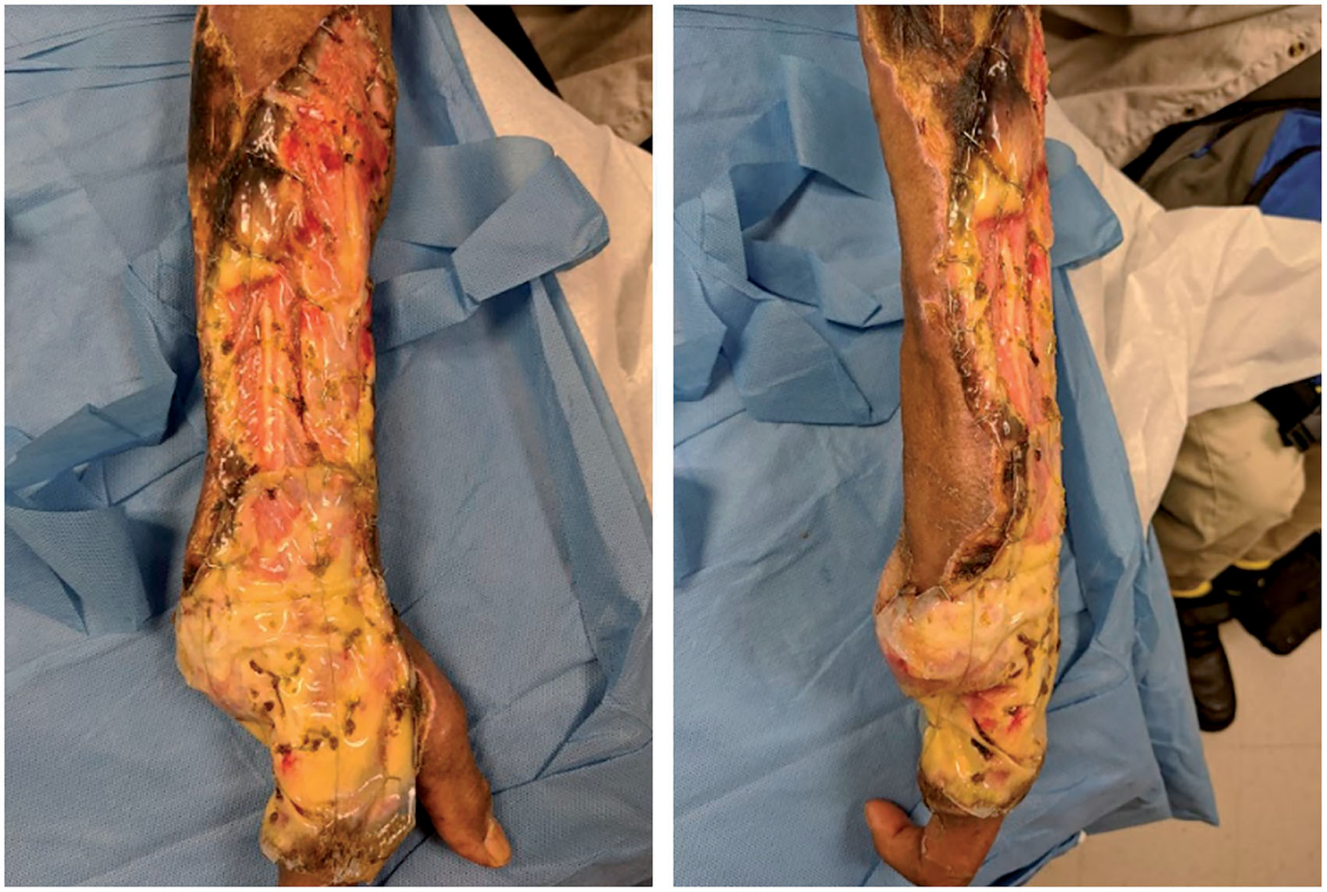
Meshed Integra placement.

**Figure 5.**
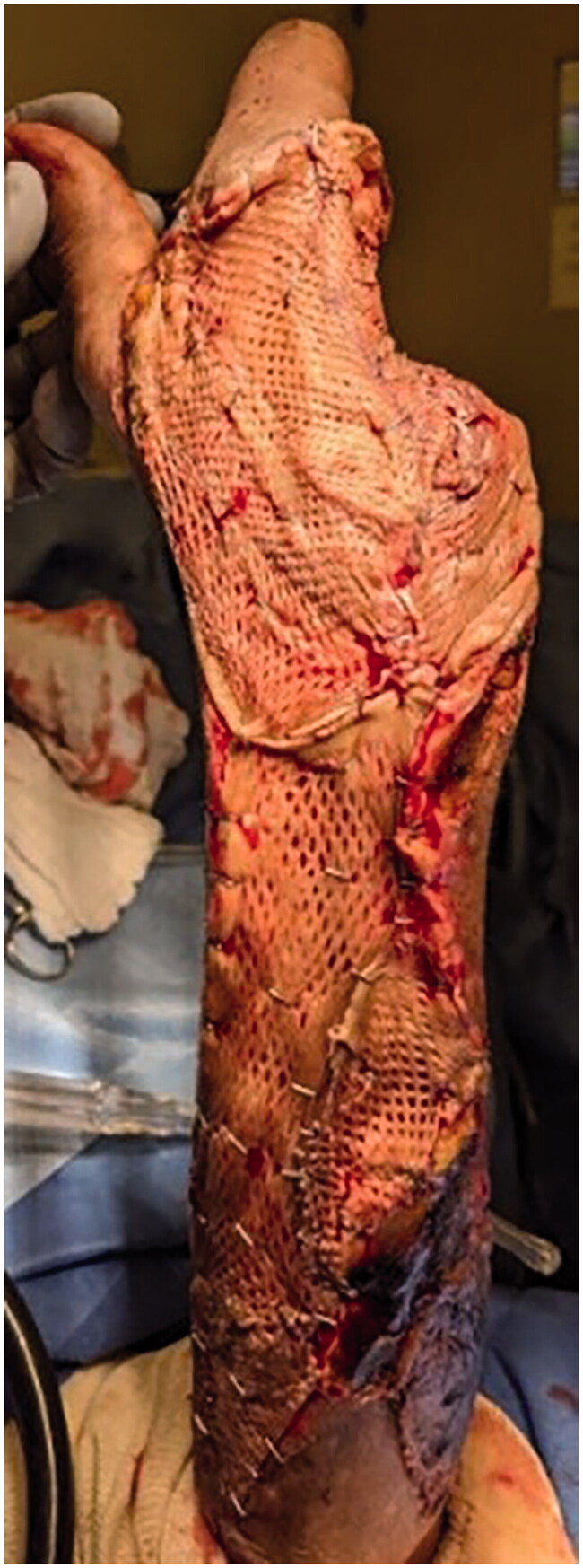
Split thickness skin graft placement.

The patient was taken to surgery by the hand surgery service within a few hours after presenting to the ED. The surgery was performed in a negative pressure room. Adequate personal protective equipment was utilized by the surgical team (Figures 2–5).

The ring and small fingers were found to be black, necrotic and non-viable. Ray amputations of the ring and small fingers were performed. Extensive debridement and removal of all necrotic tissues of the hand and the dorsal compartment of the forearm was performed. Foul smelling dish washer fluid suggestive of necrotizing fasciitis was evident. Biopsy and intra-operative cultures were obtained. Cultures demonstrated infection with Staph aureus and moderate group A Streptococcus pyogenes. Biopsy revealed extensive necrosis and inflammation of tissues.

The patient was admitted in the MICU for COVID-19, septic shock and his underlying uncontrolled multiple medical problems. Patient was treated with Azithromycin (Zithromax) and Hydroxychloroquine (Plaquenil) for COVID-19. He was treated with Piperacillin-Tazobactam for his necrotizing fasciitis.

On day two of admission, a repeat debridement was performed. Extensive necrosis of the middle finger was identified and a ray amputation of the middle finger was performed.

In the following days, the patient underwent repeat debridements of his wound, wound VAC and Integra pre-meshed bilayer wound matrix placement (Integra, LifeSciences Corporation, LLC). He demonstrated continued clinical improvement. His WBC, ESR and CRP down trended. Vasopressors were discontinued. He was transferred from the MICU to the medical floors.

Throughout the course of his admission, the patient did not require intubation for COVID-19 and he was treated with nasal oxygen and medications for COVID-19.

On week 3 of his admission, the patient’s COVID-19 PCR was negative. His wound showed early granulation and no evidence of infection. Week 3 following his admission, the patient was discharged to a sub-acute rehabilitation facility.

**Figure 6.**
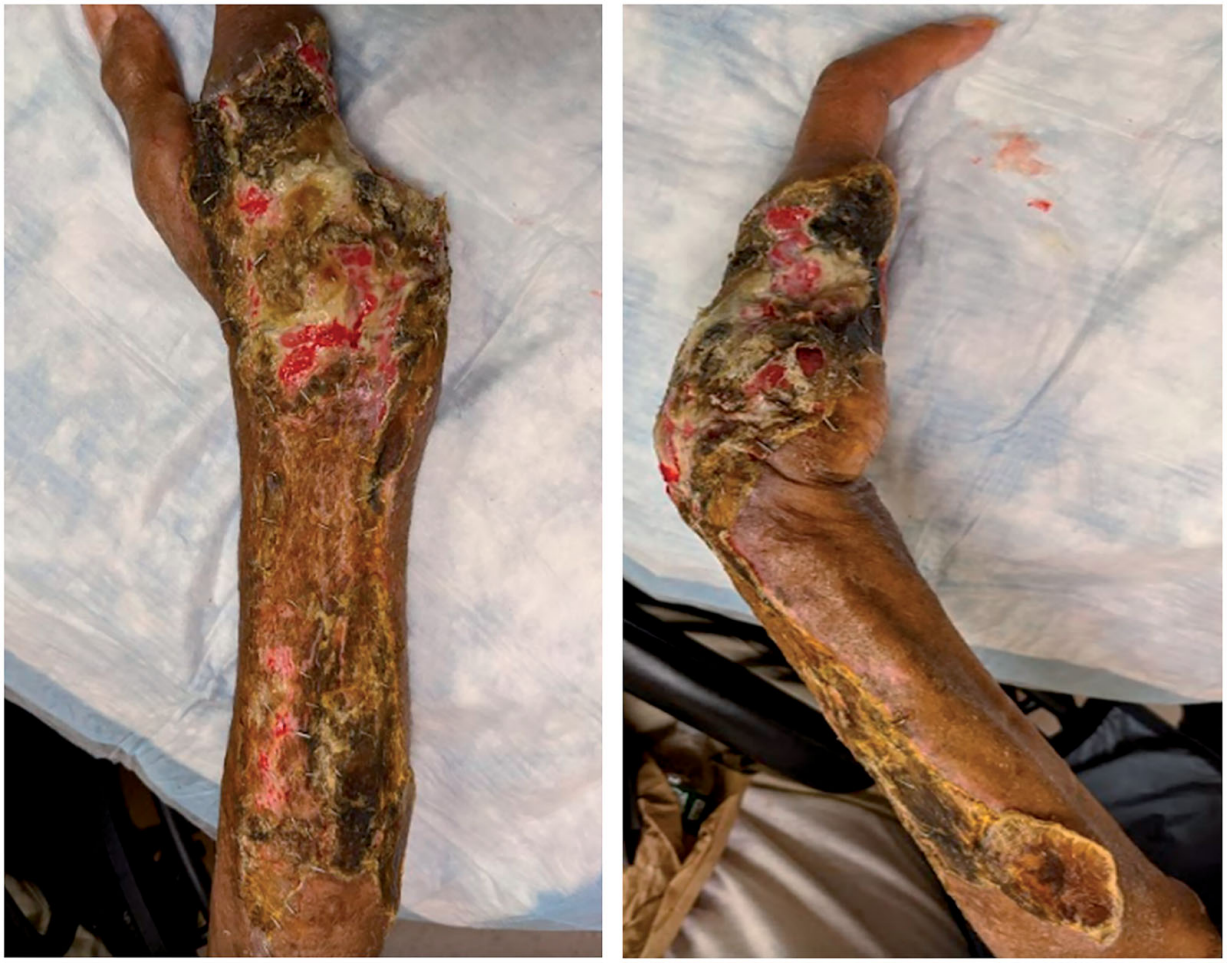
Two weeks after split thickness skin graft placement.

Split-thickness skin graft (12/1000th of an inch) from the thigh was performed in the following days. At final follow up at 3 months (Figure 6), the wound had healed completely with 100 percent take of the skin graft. The patient did not want to undergo formal hand therapy and preferred to perform exercises himself. At the time of his last evaluation at 3 months, the patient reported good function of his wrist and his remaining fingers.

**Figure 7.**
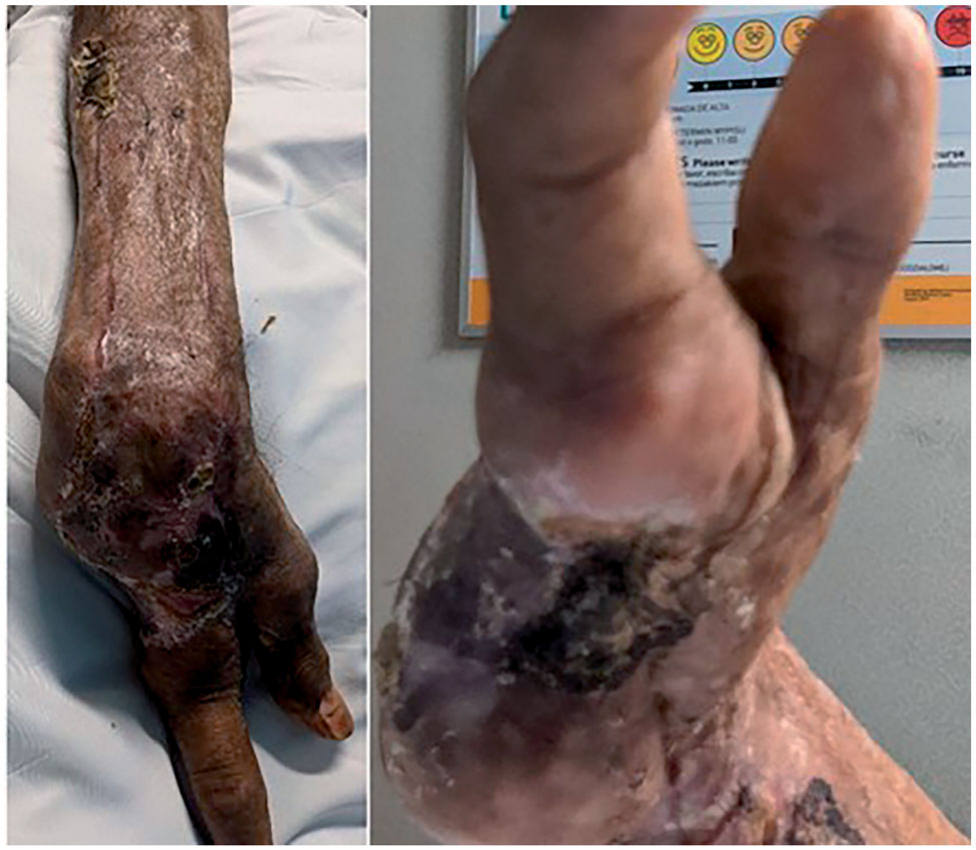
Final follow up at 3 months.

The patient subsequently did not follow up with hand surgery. Eight months after his initial admission, the patient was hospitalized at a different institution for his medical problems and passed away from post COVID-19 related lung changes and renal failure (Figure 7).

## Discussion

Necrotizing fasciitis can be a life-threatening condition affecting the upper extremity associated with high rates of morbidity and mortality [7,8]. We were aggressive in our approach in surgically managing the patient’s necrotizing fasciitis. Since the patient had multiple uncontrolled medical co-morbidities as well as COVID-19, such an approach was limb as well as life-saving.

Ischemic complications of the upper extremity have been reported in literature in patients with COVID-19 [2,3,5]. This has been associated with hyper-coagulability that might result in the setting of COVID-19 [2,3]. Of note, an arterial duplex of our patient’s upper extremity revealed patent arterial flow. This is similar to the findings in the previous reported case series of digital ischemia in COVID-19 patients [5]. The hyper-coagulopathic state from the patient’s COVID-19 likely contributed to the development and progression of his finger gangrene. In COVID-19 patients, elevated D-dimers [1–3] have been reported to be an indicator of a hyper-coagulable state and are a marker of severity of the disease. Our patient also presented with elevated D-dimer levels.

At the time of his presentation, the patient had several abnormal blood parameters. Recent studies have shown that COVID-19 can result in elevated WBC, D dimer values, ESR and CRP [9]. However, the elevated parameters in our patient could have resulted from covid, necrotizing fasciitis or his uncontrolled medical problems.

In a recent review of 42 patients with COVID-19 and Acute Limb Ischaemia (ALI), Wohlauer et al. [10] found that the upper extremity was involved in 14% of the patients. Age > 60 years, hypertension, peripheral vascular disease and diabetes were found to be risk factors for the development of Acute Limb Ischaemia. Interestingly, the authors found that while ALI may be seen in patients with severe COVID-19, it is also being recognized to occur in patients with even mild to moderate COVID-19 symptoms. In their review, ALI has also been an initial presenting symptom of COVID-19 in the absence of respiratory symptoms. The patient presented in this case report developed ischaemia and gangrene in his finger insidiously. He did not recall any trauma or other inciting factors. The patient had only mild to moderate COVID-19 pulmonary symptoms. These findings are similar to the findings of Wohlauer et al. in their review.

In the setting of the patient’s several uncontrolled medical conditions, it might be difficult to attribute the patient’s infection entirely to COVID-19. However, we hypothesize that the patient’s COVID-19 and its associated hyper-coagulopathic manifestations likely contributed to either the onset and/or evolution of the patient’s disease process.

The novelty of the case report lies in that there is no current literature on the onset and disease progression of necrotizing fasciitis in the setting of COVID-19. This is the first case report being reported on this combination of life- threatening infections.

Further research is needed to understand the ischaemic and secondary infectious complications of COVID-19. We believe that this case report will add to existing knowledge of the patho-mechanism and disease progression of hand ischaemia and infections in the setting of a patient with concomitant COVID-19.

## Conclusion

There are no current reports in existing literature about necrotizing fasciitis in a patient with COVID-19. In an elderly patient, both COVID 19 and necrotizing fasciitis are extremely life-threatening and our patient presented to us with both conditions. In addition, our patient also presented with several uncontrolled medical co-morbidities. This case illustrates that expeditious multi- disciplinary management in such patients may result in good outcomes.

There is currently a paucity of literature on COVID-19 and its various manifestations. Our understanding of its various presentations, specifically in relation to hypercoagulopathy and limb ischaemia is still evolving. We believe that this case report will add to the currently evolving knowledge of COVID-19 and its manifestations.
